# Adherence to Concurrent Tuberculosis Treatment and Antiretroviral Treatment among Co-Infected Persons in South Africa, 2008–2010

**DOI:** 10.1371/journal.pone.0159317

**Published:** 2016-07-21

**Authors:** Ernesha Webb Mazinyo, Lindsay Kim, Sikhethiwe Masuku, Joey L. Lancaster, Ronel Odendaal, Margot Uys, Laura Jean Podewils, Martie L. Van der Walt

**Affiliations:** 1 Tuberculosis HIV/AIDS Treatment Support and Integrated Therapy (THAT’SIT), Johannesburg, South Africa and Foundation for Professional Development, Pretoria, South Africa; 2 Epidemic Intelligence Service, Centers for Disease Control and Prevention, Atlanta, Georgia, United States of America; 3 Division of Tuberculosis Elimination, Centers for Disease Control and Prevention, Atlanta, Georgia, United States of America; 4 Tuberculosis Epidemiology and Intervention Research Unit, South African Medical Research Council, Pretoria, South Africa; Johns Hopkins Bloomberg School of Public Health, UNITED STATES

## Abstract

**Background:**

Adherence to tuberculosis (TB) treatment and antiretroviral therapy (ART) reduces morbidity and mortality among persons co-infected with TB/HIV. We measured adherence and determined factors associated with non-adherence to concurrent TB treatment and ART among co-infected persons in two provinces in South Africa.

**Methods:**

A convenience sample of 35 clinics providing integrated TB/HIV care was included due to financial and logistic considerations. Retrospective chart reviews were conducted among persons who received concurrent TB treatment and ART and who had a TB treatment outcome recorded during 1 January 2008–31 December 2010. Adherence to concurrent TB and HIV treatment was defined as: (1) taking ≥80% of TB prescribed doses by directly observed therapy (DOT) as noted in the patient card; and (2) taking >90% ART doses as documented in the ART medical record during the concurrent treatment period (period of time when the patient was prescribed both TB treatment and ART). Risk ratios (RRs) and 95% confidence intervals (CIs) were used to identify factors associated with non-adherence.

**Results:**

Of the 1,252 persons receiving concurrent treatment, 138 (11.0%) were not adherent. Non-adherent persons were more likely to have extrapulmonary TB (RR: 1.71, 95% CI: 1.12 to 2.60) and had not disclosed their HIV status (RR: 1.96, 95% CI: 1.96 to 3.76).

**Conclusions:**

The majority of persons with TB/HIV were adherent to concurrent treatment. Close monitoring and support of persons with extrapulmonary TB and for persons who have not disclosed their HIV status may further improve adherence to concurrent TB and antiretroviral treatment.

## Introduction

South Africa remains at the center of the HIV and tuberculosis epidemics [1]. Among high TB burden countries, South Africa had an incidence rate of 509 new cases co-infected with TB and HIV (TB/HIV) per 100,000 persons in 2014, leading to a substantial number with TB/HIV who require concurrent treatment for both diseases [2].

Prior to 2009, South African guidelines reserved initiation of antiretroviral therapy (ART) for adults with CD4 counts < 200/mm^3^ [3]. In 2010, revised guidelines outlined eligibility for ART among persons with TB/HIV to include those with CD4 cell count ≤ 350 cells/mm^3^ and those with multidrug-resistant or extensively drug-resistant TB (MDR-TB or XDR-TB), irrespective of CD4 cell count [4]. The World Health Organization (WHO) published recommendations in the same year promoting the initiation of ART for all persons with HIV/TB co-infection, irrespective of the CD4 cell count [5].

Adherence to both TB treatment and ART is a key determinant of TB/HIV treatment outcomes, including lower morbidity and mortality [6–7], and essential to minimize the emergence of both TB and ART drug resistance [8]. Previous studies have identified HIV beliefs, stigma associated with HIV disclosure, food and transportation costs, substance use, pill burden, adverse drug events, and poor communication with healthcare workers (HCWs) as factors associated with ART non-adherence [9–10]. Similar factors have been cited as increasing risk for non-adherence to tuberculosis treatment [11–12]. A South African study reported that patient perception of poor attitudes among HCWs and the patient changing residence during TB treatment were both independently associated with TB treatment interruption among both new and re-treatment patients [11]. A qualitative study in Brazil, another high burden country, found that TB non-adherence was associated with low belief in TB curability, low belief in severity of TB in the presence of HIV infection, and low levels of support from family and HCWs [12]. Qualitative studies have also cited side effects, pill burden, substance abuse, economic constraints, food security, and stigma as risk factors of non-adherence to concurrent TB treatment and ART [13–16].

While adherence has been studied for TB and HIV individually, factors influencing adherence to concurrent TB treatment and ART in South Africa are less well documented. To our knowledge, a small number of studies [7, 17–19] have examined concurrent ART and TB treatment in South Africa under programmatic conditions. One study found a high level of adherence [17], while one cross-sectional study found high levels of non-adherence (42.4%) among patients after 1 month of concurrent treatment [18]. This latter study also found that poverty, having ≥ 1 co-morbid condition, being at risk for alcohol misuse, and having a partner with HIV were significant factors relating to non-adherence [18]. A qualitative study by Daftary et al. examined adherence to ART and second-line TB treatment in individuals co-infected with HIV and MDR-TB or XDR-TB and found that ART was preferred over TB treatment because of greater tolerability, lower pill burden, and feeling committed to ART [19]. Lastly, Uyei et al. found that integrated TB and ART service delivery and the delivery of TB and HIV care by the same clinical team reduced the odds of death [7].

As the number of TB/HIV co-infected patients increase in South Africa, it is important to examine factors related to adherence in this population to maximize the effectiveness of both treatments. Therefore, we conducted a study among persons co-infected with TB/HIV to determine the level of adherence to concurrent administration of ART and TB treatment and to identify risk factors for non-adherence in two South African provinces during 2008–2010.

## Methods

### Setting and Study Population

The study was conducted in two districts of North West Province (NW) (Dr. Ruth S. Mompati District and Dr. Kenneth Kaunda District) and one district in Western Cape Province (WC) (Eden District). During the study period, South Africa had the highest TB incidence globally (997 per 100,000 persons) [20], with incidences of 800 and 965 per 100,000 persons in NW and WC provinces, respectively [21]. HIV prevalence in South Africa was 10.9%, while prevalence in NW and WC provinces was 11.3% and 3.8%, respectively [22]. Nationally, HIV co-infection among TB patients was reported to be 67% [20].

The TB, HIV/AIDS, Treatment Support and Integrated Therapy (THAT’SIT) program was established in 2006 with the explicit goal of supporting the National Department of Health (NDoH) in providing a comprehensive range of integrated services for persons with TB and HIV, including, but not limited to, integrated patient records, implementing community-based care of TB/HIV co-infected patients, and establishing a community referral network that can trace patients who did not keep clinic appointments, collection and delivery of patients’ medications, and encouraging HIV and TB screening [23]. A convenience sample of 35 THAT’SIT-supported NDoH clinics, 23 in NW and 12 in WC, were selected for the study due to financial and logistic considerations. THAT’SIT maintains an electronic database that serves as the primary source for TB and HIV patient-level information at the supported clinics [24]. The study population included all adults (≥18 years of age) with smear-positive TB (new and retreatment) identified through the THAT’SIT database to be taking concurrent TB treatment and ART for at least one month during 1 January 2008–30 June 2010. Patients could have already been on concurrent treatment before the study period. Data collection was censored at 31 December 2010, which meant that the last patient eligible for the study was allowed 6 months to complete first-line TB treatment (i.e., standard 4-drug regimen). Patients were excluded if they transferred outside of the study facilities, were documented to have MDR-TB, or were missing a recorded TB treatment outcome by 31 December 2010.

### Study Design and Data Collection

A retrospective chart review of the TB and HIV data sources in THAT’S’IT-supported NDoH clinics was conducted. These data sources included the South Africa National TB Control Programme Blue Card (considered the primary medical chart for TB patients), the government’s ART wellness form, and THAT’SIT patient forms. The latter were developed to supplement the government-issued blue card and wellness forms and served as the THAT’S’IT records for TB/HIV integrated care. We abstracted data on a standardized questionnaire that included patient socio-demographics, medical treatment history (e.g. pregnancy, chronic diseases, and mental health), clinical parameters of their current episode of TB disease and HIV, prescribed TB and HIV treatment regimens and treatment adherence, and the final TB treatment outcome. Collected data also included WHO clinical stage for HIV, CD4 cell count and viral load results, ART start and end dates, adverse events, toxicities, substance use, and psychosocial factors (e.g., disclosure, having a treatment supporter, support group attendance, home visits, etc.). Trained healthcare workers abstracted and recorded all data at the clinic. Double entry data capturing was completed at the South African Medical Research Council regional office in Pretoria.

### Study Definitions

Concurrent treatment was defined as a TB/HIV co-infected patient receiving simultaneous TB treatment and ART for a minimum of one month. Adherence to concurrent treatment was defined as taking ≥80% of TB doses by directly observed therapy (DOT, i.e. taking doses daily at the clinic under supervision of a clinician or at a mutually agreed upon community location under supervision of a community treatment supporter) as recorded in the TB Blue Card and taking > 90% of ART doses as documented in the ART medical record during the concurrent treatment period. Adherence percentage cut-offs were determined using previous publications [25–27]. The concurrent treatment period occurred at any time during 1 January 2008–31 December 2010. Patients were defined as non-adherent if they did not meet the criteria for adherence mentioned above.

### Analysis

Descriptive statistics were used to describe the demographic, clinical and psychosocial characteristics of all patients included in the study, and by category of adherence (adherent or non-adherent). The Pearson’s chi square test and the Fisher’s exact test were used for comparisons by adherence category. To examine factors associated with non-adherence, we examined each variable individually and calculated risk ratios (RR) and 95% confidence intervals (CI). We then developed a multivariate model using variables with a *P* <0.20 on bivariate analysis or otherwise informed as important through previous studies. Stepwise regression was performed, and in the final model, factors with a *P* <0.05 were considered statistically significant. All analyses were conducted using STATA12 (Stata Corp LP, College Station, TX, USA).

### Ethical Approval

This study was reviewed and approved by the Institutional Review Boards of the South African Medical Research Council and the US Centers for Disease Control and Prevention. Ethical approval was obtained from the two provinces and the respective clinics prior to the study. This study did not require informed consent because the data was based on existing programmatic information and retrospectively abstracted from medical charts. Patient records and information was anonymized and de-identified prior to analysis.

## Results

**Fig 1:**
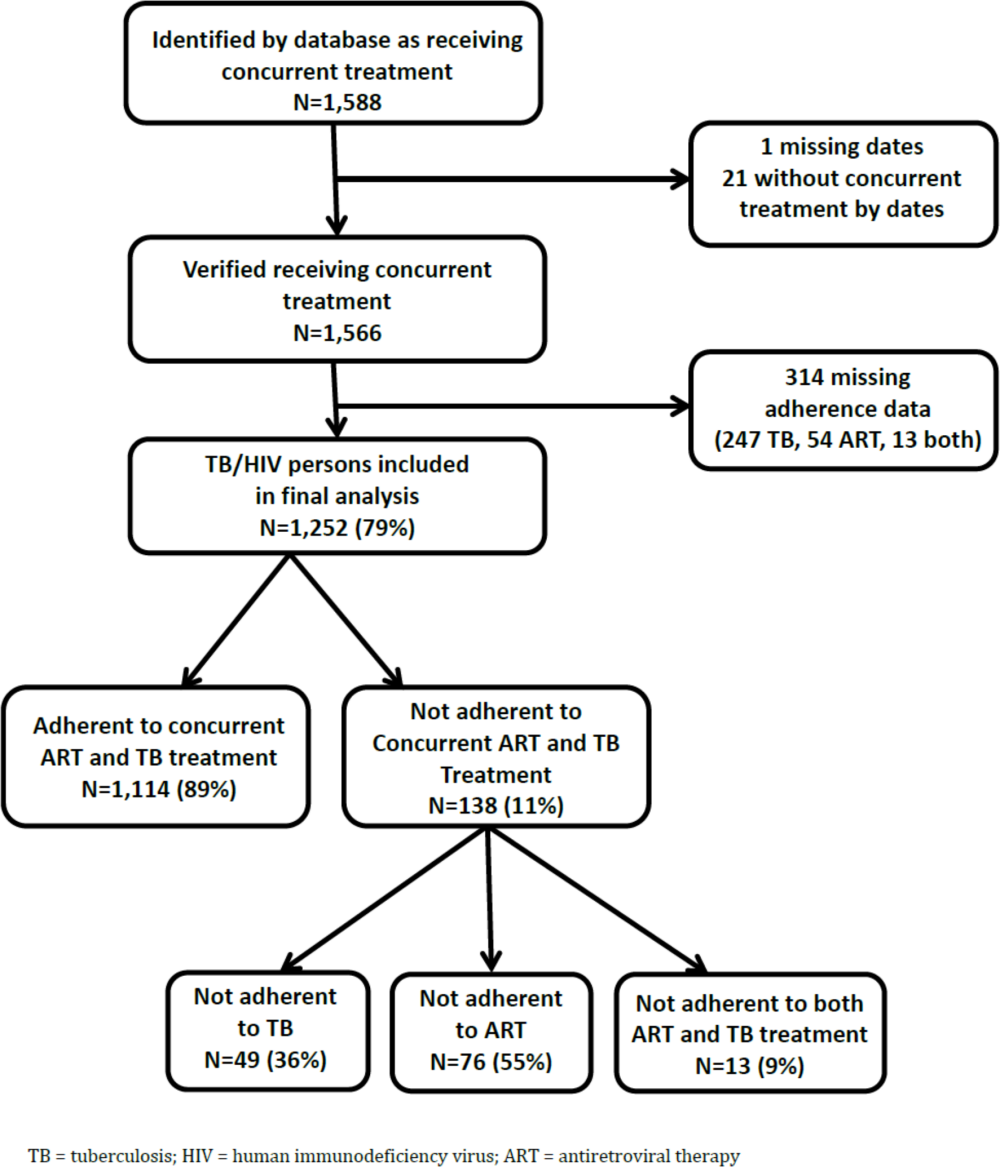
Study Population Included in TB/HIV Concurrent Treatment Analysis, 2008–2010.

A total of 1,588 TB/HIV patients listed in the THAT’SIT database were identified as potentially receiving concurrent ART and TB treatment during January 2008–December 2010. Twenty-two patients were excluded as they were not receiving concurrent ART and TB treatment. From the remaining 1,566 patients, 314 had missing TB or HIV adherence data and were excluded from the study, resulting in 1,252 patients included in the final analyses (Fig 1). Overall, 11% (n = 138) of patients were not adherent to concurrent treatment. Among these, 55.1% (n = 76) were adherent to TB treatment only, 35.5% (n = 49) were adherent to ART only, and 9.4% (n = 13) were not adherent to either treatment (Fig 1).

Of the 1,252 persons receiving concurrent TB/HIV treatment, 45.4% were aged 30–39 years, 56.0% (n = 698) were women, and 21.2% (n = 232) were employed (Table 1). Among the 915 persons with measured height and weight, 42% of patients were underweight according to body mass index. Mental disorder was reported by 6.4% (n = 63) of 985 patients, while more patients reported substance use (25.6%, 200/782).

**Table 1:** Demographic, clinical, and social characteristics of patients receiving concurrent TB/HIV treatment in selected THAT’SIT clinics, South Africa, 2008–2010 (N = 1,252). BMI = body mass index; TB = tuberculosis; IRZE = isoniazid, rifampin, pyrazinamide, ethambutol; DOT = directly observed therapy; WHO = World Health Organization; HIV = human immunodeficiency virus; ARV = antiretroviral; D4T/3TC/EFV = stavudine, lamivudine, efavirenz; D4T/3TC/NVP = stavudine, lamivudine, nevirapine.

Characteristic	Total n (%†)
Age at concurrent treatment start, years	
<30	245 (19.6)
30–39	568 (45.4)
40–49	304 (24.3)
≥50	135 (10.8)
Sex (n = 1247)	
Female	698 (56.0)
Male	549 (44.0)
Employment status (n = 1097)	
Employed	232 (21.2)
Unemployed	865 (78.9)
BMI at start of concurrent treatment (kg/m^2^) (n = 915)	
Underweight (<18.5)	391 (42.7)
Normal (18.5–24.9)	433 (47.3)
Overweight/Obese (≥25)	91 (10.0)
Mental disorder (n = 985)	
No	922 (93.6)
Yes	63 (6.4)
Substance use (n = 782)	
No	582 (74.4)
Yes	200 (25.6)
TB patient category (n = 1247)	
New	928 (74.4)
Retreatment	319 (25.6)
TB site of disease	
Pulmonary	1092 (87.2)
Extrapulmonary	160 (12.8)
DOT for TB treatment	
DOT full treatment course	1186 (94.7)
DOT intensive phase only	26 (2.1)
DOT continuation phase only	39 (3.1)
No DOT	1 (0.1)
WHO clinical HIV stage	
Stage 1	72 (7.3)
Stage 2	100 (10.1)
Stage 3	530 (53.6)
Stages 4	287 (29.0)
ARV regimen (n = 1146)	
1a (D4T/3TC/EFV)	1072 (93.5)
1b (D4T/3TC/NVP)	74 (6.5)
CD4 at concurrent treatment start, (units) (n = 1011)	
≤200	789 (78.0)
>200	222 (22.0)
Disclosed HIV status‡ (n = 1046)	
Yes	1006 (96.2)
No	40 (3.8)
Number of toxicities during the first 2 months of concurrent treatment	
None	710 (56.7)
1	274 (21.9)
≥2	268 (21.4)

†Percentages reflect the proportion among participants with a recorded value for each given variable (missing were excluded from percent), so totals may not add to 1252.

‡Variable indicates whether or not the patient reported disclosing his or her HIV status to a friend or family member.

The majority of patients (74.4%; n = 928) were classified as a new TB case, and 87.2% (n = 1092) had pulmonary TB and received either the Category 1 TB regimen (i.e., 2 months of isoniazid, rifampicin, pyrazinamide, and ethambutol given daily, followed by isoniazid and rifampicin given daily for 4 months) or Category 2 TB regimen (i.e., 2 months of isoniazid, rifampicin, pyrazinamide, and ethambutol and streptomycin injections given daily, followed by IRZE only in the third month, then by IRE during the continuation phase of 5 months) [28]. The majority of patients received DOT for the full duration of TB treatment (n = 1186, 94.7%). Also, 82.6% of patients were at an advanced HIV clinical stage (i.e., WHO clinical HIV stage 3 or 4) at the onset of concurrent treatment, and 78% had a CD4 cell count <200 cells/mm^3^. Lastly, 43.3% of TB/HIV patients experienced one or more toxicities during the first two months of concurrent treatment (Table 1).

On bivariate analysis, patients with extrapulmonary TB disease had a significantly increased risk for non-adherence (*P* = 0.001) (Table 2). Male gender (*P* = 0.09), unemployment (*P* = 0.08), and patients who did not disclose their HIV status to at least one friend or family member (*P* = 0.06) were borderline significant (Table 2); all were included in the final multivariate model, however, since *P* <0.20.

**Table 2:** Bivariate relationship between characteristics of persons receiving concurrent TB/HIV treatment in selected THAT’SIT clinics and adherence to treatment (N = 1,252). BMI = body mass index; TB = tuberculosis; HRZE = isoniazid, rifampin, pyrazinamide, ethambutol; HRZES = isoniazid, rifampin, pyrazinamide, ethambutol, streptomycin; DOT = directly observed therapy; WHO = World Health Organization; HIV = human immunodeficiency virus; ARV = antiretroviral; D4T/3TC/EFV = stavudine, lamivudine, efavirenz; D4T/3TC/NVP = stavudine, lamivudine, nevirapine.

Characteristic	Adherent to concurrent ART and TB treatment, N = 1114 n (%)	Not adherent to concurrent ART and TB treatment, N = 138 n (%)	RR (95% CI)	*P* value
Age at concurrent treatment start, years				
<30	212 (86.5)	33 (13.5)	ref	
30–39	511 (90.0)	57 (10.0)	0.75 (0.50–1.11)	0.15
40–49	267 (87.8)	37 (12.2)	0.90 (0.58–1.40)	0.65
≥50	124 (91.9)	11 (8.1)	0.60 (0.32–1.16)	0.13
Sex				
Female	630 (90.3)	68 (9.7)	ref	
Male	479 (87.2)	70 (12.8)	1.31 (0.96–1.79)	0.09
Employment status				
Employed	213 (91.8)	19 (8.2)	Ref	
Unemployed	758 (87.6)	107 (12.4)	1.51 (0.95–2.41)	0.08
BMI at start of concurrent treatment (kg/m^2^)				
Normal (18.5–24.9)	368 (85.0)	45 (15.0)	ref	
Underweight (<18.5)	347 (88.7)	44 (11.3)	1.08 (0.73–1.60)	0.69
Overweight/Obese (≥25)	86 (94.5)	5 (5.5)	0.53 (0.22–1.29)	0.16
Mental disorder				
No	821 (89.0)	101 (11.0)	ref	
Yes	53 (84.1)	10 (15.9)	1.45 (0.80–2.63)	0.22
Substance use				
No	529 (90.9)	53 (9.1)	ref	
Yes	179 (89.5)	21 (10.5)	1.15 (0.71–1.86)	0.56
TB patient category				
New	826 (89.0)	102 (11.0)	ref	
Retreatment	283 (88.7)	36 (11.3)	1.03 (0.72–1.47)	0.89
TB site of disease				
Pulmonary	984 (90.1)	108 (9.9)	ref	
Extrapulmonary	130 (81.3)	30 (18.7)	1.90 (1.31–2.74)	0.001
DOT for TB treatment				
DOT full treatment course	1053 (88.8)	133 (10.2)	ref	
DOT intensive phase only	22 (84.6)	4 (15.4)	1.37 (0.55–3.43)	0.50
DOT continuation phase only	38 (97.4)	1 (2.6)	0.23 (0.03–1.59)	0.14
No DOT	1 (100.0)	0 (0.0)	- -	- -
WHO HIV stage				
Stage 1	65 (90.3)	7 (9.7)	ref	
Stage 2	91 (91.0)	9 (9.0)	0.93 (0.36–2.37)	0.87
Stage 3	474 (89.4)	56 (10.6)	1.09 (0.52–2.29)	0.83
Stage 4	254 (88.5)	33 (11.5)	1.18 (0.55–2.56)	0.67
ARV regimen				
1a (D4T/3TC/EFV)	952 (88.8)	120 (11.2)	Ref	
1b (D4T/3TC/NVP)	67 (90.5)	7 (9.5)	0.85 (0.41–1.74)	0.65
CD4 at concurrent treatment start, (units)				
≤200	708 (89.7)	81 (10.3)	ref	
>200	193 (86.9)	29 (13.1)	1.27 (0.86–1.89)	0.23
Disclosed HIV status				
Yes	899 (89.4)	107 (10.6)	ref	
No	32 (80.0)	8 (20.0)	1.88 (0.99–3.58)	0.06
Number of toxicities during the first 2 months of concurrent treatment				
None	631 (88.9)	79 (11.1)	ref	
1	242 (88.3)	32 (11.7)	1.05 (0.71–1.54)	0.81
≥2	241 (89.9)	27 (10.1)	0.91 (0.60–1.37)	0.64

In the final multivariate model adjusted for age, having extrapulmonary TB disease (RR = 1.71, 95% CI 1.12 to 2.60) and not disclosing HIV status to at least one friend or family member (RR = 1.96, 95% CI 1.02 to 3.76) remained independent risk factors for non-adherence to concurrent TB treatment and ART (Table 3). No other variables considered in the multivariate model were significant.

**Table 3:** Multivariate analysis of the relationship between characteristics of persons receiving concurrent TB/HIV treatment in selected THAT’SIT clinics and non-adherence to concurrent TB and HIV treatment (N = 1252). TB = tuberculosis, HIV = human immunodeficiency virus.

Characteristic	RR (95% CI)	*P* value
Age at concurrent treatment start, years	0.99 (0.97–1.01)	0.34
TB site of disease		
Pulmonary	ref	
Extrapulmonary	1.71 (1.12–2.60)	0.01
Disclosed HIV status		
Yes	ref	
No	1.96 (1.02–3.76)	0.04

## Discussion

Our study found that the majority of patients with TB/HIV were adherent to concurrent treatment at THAT’SIT supported clinics and that patients were more frequently adherent to TB treatment compared to ART. Factors associated with non-adherence included extrapulmonary TB disease and not having disclosed HIV status to at least one friend or family member. There were no significant differences in HIV clinical and treatment characteristics between the adherent and non-adherent groups. More than 80% of the study population were at an advanced HIV clinical stage and CD4 cell counts were similar between adherent and non-adherent groups. Such low rates of non-adherence to concurrent treatments suggest that patients may be able to successfully manage concurrent treatment in resource-limited settings.

Contrary to previous literature [13], HIV disease severity did not play into the factors that influence adherence in our study, suggesting that other factors might be more important in adherence.

We found that extrapulmonary TB influenced adherence negatively, which aligns with findings from another study [29]. In the context of HIV, patients with extrapulmonary TB may have advanced immune suppression, as they are usually sicker patients with advanced HIV disease, and thus, they are at higher risk for poor treatment outcomes [30]. In the present study, there were no remarkable differences in WHO stage or CD4 count between patients with TB and extrapulmonary TB at the time of initiating treatment; however, it is possible that these indices changed over the course of treatment. Studies have also shown that patients with extrapulmonary TB experience longer treatment delays than patients with pulmonary TB [31]. Further research is needed to fully explore the underlying reasons for non-adherence in HIV patients with extrapulmonary TB.

Our analysis also revealed that non-adherent persons were more likely to have not disclosed their HIV status to at least one friend or family member. Several studies have found that non-disclosure is a barrier to retention in care [32–35]. Provision of psychosocial support that encourages patients to disclose may reduce non-adherence, as patients who disclose their HIV status to relatives and close friends experience less stigma and discrimination to obtain and take drugs. In fact, disclosing one’s HIV status was found to be essential for receiving social support like reminders to take pills, transportation and food costs, and emotional backing [32]. Programs could consider closely monitoring patients who have not disclosed HIV status to a family member or friend when first initiating ART or TB treatment, or counsel them to disclose, as this could be a signal to healthcare providers of the diminished likelihood of adherence to the complicated regimens for HIV and TB.

Unlike research that shows a preference for ART over TB treatment [19], we found that patients were more frequently adherent to TB treatment compared to ART. This finding might be due to the shorter duration that a patient must take TB treatment compared to life-long ART but may also suggest that the preference for ART may be diminished when patients are receiving integrated TB/HIV treatment support. With South Africa having a growing burden of drug-resistant TB, it is important to note that higher adherence to TB treatment as found in our study may lend credence to programs like THAT'SIT which employed more aggressive efforts to identify, treat and support TB patients who were found to be HIV positive. THAT'SIT integrated the empowerment approach of the HIV program by offering patient education, treatment literacy, adherence counseling and support for effective self-management with the regular monitoring of the TB program that required routine treatment supervision [23]. Though this study precludes us from drawing direct associations between THAT'SIT and adherence outcomes, our high co-adherence finding supports evidence that urges review of TB and HIV treatment provision and the disjuncture between the treatment supervision approach of the TB program and the patient empowerment design of the HIV program [36].

Historically, HIV and TB have been treated in separate programs and facilities. TB clinics have been slow to implement HIV care, including the introduction of ART, which has resulted in the establishment of separate HIV clinics that often do not treat TB co-infection [37–38]. Our findings provide evidence to support integration of TB and HIV services in primary health care settings as adherence rates in our study are higher than those found in previous studies in Africa [13,15,16] and consistent with findings of studies with smaller study populations in KwaZulu-Natal, South Africa receiving integrated care [6, 17].

There were several limitations to our study. First, this was a convenience sample of THATS’IT-supported clinics, and it is possible that these clinics performed differently compared to other THATS’IT-supported or other integrated TB/HIV clinics; however, we did attempt to capture both clinics in rural and urban settings. Second, we relied on routinely collected data for TB and for ART (both standard DoH forms and THAT’S IT databases) for this evaluation, which was not initially intended for research purposes. We found variability in the ART forms used across provinces and clinics, which caused difficulty in the standardization of some data variables. However, while there has been consistent documentation of the inaccuracy of standard information collected for TB and surveillance purposes [39], previous evaluations have reported almost perfect levels of completeness and accuracy in THAT’S IT databases, as they have dedicated staff and utilize supplemental information beyond the standard DoH forms [24]. Though data were extracted and validated across TB and HIV referencing sources for the study, data quality and completeness remains a matter of concern in the routine functioning of public health facilities in South Africa. We were missing adherence information on 314 patients in the original cohort identified as initiating concurrent treatment during the study period (Fig 1). After performing a sensitivity analysis, it is reasonable that the missing observations are unlikely to alter the study conclusions because the 314 have similar distributions in their characteristics as the final 1252 included in the study analysis. If a large proportion of these patients had missing outcomes due to default or noncompliance, however, then our findings would overestimate adherence to concurrent treatment. Third, the study lacked direct, validated measures of adherence as confirmed by previous studies [40]. Fourth, factors identified in previous literature [41], including adverse events, substance use, and disease severity were not associated with adherence in this study population, but this may be due to small sample size in the non-adherent group and missing data. Future research should investigate the impact of patterns of treatment interruption on adherence. Fifth, this study collected limited data associated with the social, economic, treatment, and illness-related dimensions of adherence [42] because of data unavailability; therefore, the study was restricted in its ability to measure the impact that patient-provider interaction, health system characteristics, and patient-level factors affect adherence. Lastly, because those patients that were transferred out of the facility were excluded, and these patients might have poorer adherence, we might have introduced some bias towards better adherence in this study. While the results of this study apply to THAT'SIT supported clinics and should only be generalized to other TB/HIV integrated clinics with caution, the study offers insight to factors that may affect concurrent TB/HIV treatment adherence in similar high-burden settings.

In conclusion, this study demonstrated the capacity to achieve a high level of adherence to concurrent TB and ART in an integrated TB/HIV care setting. While the implications of our findings support the current South African National Strategic Plan to scale-up initiation of concurrent TB/HIV treatment under integrated care [43], persons with extrapulmonary TB disease and those who fail to disclose their HIV status to at least one friend or family member may be at an increased risk for non-adherence to concurrent treatment and should receive close monitoring to prevent non-adherence. Future studies may further examine underlying reasons persons fail to disclose their status and the association of extrapulmonary TB disease and adherence.
